# The Hospital. Nursing Mirror

**Published:** 1898-05-21

**Authors:** 


"The Hospital" Nursing Mirror.

Being the Nursing Section of "The Hospital."

[Contributions for this Section of "The Hospital" should be addressed to the Editor, The Hospital, 28 & 29, Southampton Street, Strand, London, W.C., and should have the word "Nursing" plainly written in left-hand top corner of the envelope.]

### News from the Nursing World.

### HER MAJESTY AT NETLEY.

On Saturday the Queen spent an hour and a half at Netley Hospital, and saw all the patients, except about sixty. She left Windsor at half-past two, and arrived at Netley at half-past four. Her Majesty was in excellent health and spirits, and walked through the station to her carriage. The route to the hospital was decorated, and thousands of spectators gave her an enthusiastic greeting. Surgeon-Major-Generals Nash and Kelly accompanied the Queen as she was wheeled through the wards in which were the convalescents, and Surgeon-Major Dick as she went through those on the second floor. Piper Findlater and Private Vickery, both of Dargai renown, had the honour of receiving the Victoria Cross from Her Majesty's own hands.

### IN MEMORIAM OF THE DUCHESS OF TECK.

A wish near the heart of the late Duchess of Teck is about to be fulfilled, for the Home of Rest for Poor Women on the South Coast is now a certainty. At a meeting of the Mansion House Committee on the 13th inst. it was announced that subscriptions had been received to the amount of £9,000, and that a generous donor who wished to remain anonymous had offered to give the site, and to build the home. It is hoped that the remaining £2,000 of the £11,000 asked for by the Mansion House Committee will be forthcoming by the end of June. It will be invested as an endowment in the names of six trustees nominated by the Duchess of York, and will render the memorial independent of further aid. The home will be built at Bognor on a site next to the Victoria Convalescent Home for Women at Bognor, and will be large enough to give 130 women a fortnight's rest every year.

### FLORENCE NIGHTINGALE'S BIRTHDAY.

Miss Florence Nightingale's birthday fell on Sunday last. She was born at Florence on May 15th, 1820. Although she is now 78 years of age, and lives a retired life, she takes a keen interest in nursing topics, especially as applied to army work.

### JUSTICE TO NURSES.

The Hon. Sydney Holland, in his characteristic appeal on behalf of the London Hospital, speaking of the increased cost of the nursing, says: "Nursing cannot be done as it used to be, because, thank goodness, managers of hospitals have realised that nurses are human, and that it is not fair to work young women, however willing, for more than ten to eleven hours in wards with sick people every day of the seven."

### THE IRISH NURSES AT THE VICEROY'S.

On Wednesday and Thursday the Viceroy and Lady Cadogan entertained the hospital nurses of Dublin at a couple of garden parties at the Viceregal Lodge. The military and police bands played in the grounds and various amusements were provided for the guests. Giving the party in duplicate showed much kindly forethought on the part of the hosts, as by this means every nurse had the opportunity of enjoying their hospitality without its interfering with their duties.

### ST. JOHN'S HOUSE.

The St. John's House Nursing Institution celebrates its jubilee this year, and is issuing an appeal for subscriptions to the St. John's House Nurses' Pension Fund as a memorial of the event. The institution was founded in 1848, and its president is always the Bishop of London for the time being. The nurses are under the charge of a Lady Superior. They are trained and employed in hospitals, poor-law infirmaries, and private families. Eleven are now on the pension list, the capital of which is £3,500. Another £3,000 is asked for to place the fund on a firm financial basis.

### THE PRINCESS CHRISTIAN'S NURSING HOME.

A performance of the "Home Secretary" was recently given at the Matinée Theatre in aid of the Princess Christian's Trained Nurses' Home at Windsor. The actors and actresses were aristocratic amateurs, many of them bearing titles. They played their respective parts well, were charmingly dressed, and, as there was a full house, the affair was a success from every point of view.

### WORKHOUSE NURSING IN BELFAST.

The following are the resolutions recently approved by the Belfast Board of Guardians with regard to the workhouse nurses. The period of service is fixed at three years. No remuneration will be given during the first year, uniform and a salary of £8 will be paid the second year, and that sum increased to £14 for the third year. The Guardians reserve the right of dismissal during the period of training. Although probationers leaving before the expiration of three years will not be entitled to a certificate, they will receive a signed paper testifying the length of time they have served. At the end of three years, and after having passed satisfactory examinations, nurses will be eligible for superior positions, for which the salaries will be £25 for the first, £27 10s. for the second, and £30 for the third year's work.

### A LEGAL POINT.

A legal matter of some importance to the nursing world was decided by Mr. Justice Mathew the other day, when Miss Burrell obtained damages amounting to £83 from her landlord, Mr. Brown. Miss Burrell carries on a nursing home at 59, Weymouth Street, and having been annoyed by stuffy smells, and cases of illness occurring in her house, she caused the drains to be examined. Mr. Brown, who had previously lived in the house, let it to her on the understanding that the drainage was perfect, and he maintained that opinion in court. It is important for tenants to remember that the landlord is not responsible for the good drainage of a house unless he represents that it is in order at the time of his letting it.

### THE EDINBURGH DIAMOND JUBILEE FUND.

The handsome sum of £21,277 has been collected for this fund. At a meeting of the Executive Committee held on the 13th inst., at which the report was unanimously adopted, it was resolved to give nine-tenths towards a pavilion for the special diseases of women at the Royal Infirmary, and the remaining tenth to Queen Victoria's Jubilee Institute for Nurses.

### NURSING IN PRISONS.

Sir Matthew White Ridley has just promised that there shall be a reorganisation of the male and female hospital staffs in the larger prisons, which will give sick prisoners the privilege of being cared for by attendants trained in sick nursing. The governors of the smaller prisons have also received orders to arrange with local nursing institutions to provide nurses when necessary.

### NURSING IN A STATE HOSPITAL.

The Chief Secretary of the New South Wales Government has received a letter from Mr. A. Griffith, M.P., formulating a series of grave charges as to the treatment of the nursing staff at Coast Hospital, Little Bay, Sidney. This is a Government hospital, having a lazaret for lepers (our readers may remember an article describing it some time ago), and special accommodation for fever cases. There is a nursing staff of 30 under a matron, and the average number of patients occupying it is 204. About six months ago Parliament voted money to build and furnish additional quarters for the nurses. It decreed that the hours on duty—namely, from six a.m. to six p.m. one day, and from six a.m. to two p.m. and from six p.m. to eight p.m. on the alternate days should be shortened. Mr. Griffith says that not a stick or stone is laid for the new buildings; that the hours are now from six a.m. to six p.m. daily for seven days a week; that barely half-an-hour is allowed for meals; that inferior rations used to be supplied, and the official who was then responsible still holds his place; that as many as six nurses are crammed into one bedroom; and that probationers, having passed their examinations, are kept in inferior positions. These charges represent a state of things so atrocious that we trust it is too highly coloured, for it is impossible to imagine practical women submitting to it.

### BICYCLES NECESSITIES.

The following riddle was somewhat aptly asked and answered in a comic journal not so very long ago: "When will bicycles go out of fashion?"—"When they are recognised as a useful means of transit." Judged from this standpoint the fashionable days of the wheel are already numbered, for at the annual meeting of the Peterborough Nursing Association subscribers were urgently asked to give enough to cover expenses, leaving windfalls from concerts and entertainments to provide bicycles, wheelchairs, and other necessities. That bicycles should soon be regarded as a necessary part of nurses' equipment is probably the last thing its inventor thought of.

### CARELESS ACCOUNT KEEPING.

Much vexation of spirit would be spared if the business-like habit of keeping strict accounts were universally observed. A case in point occurred at Chesterfield lately, when Judge Smyly allowed the Nurses' Institute only £2 of a claim for £14, because no definite accounts had been kept of the extras supplied to in-patients. The invalid, the son of an engine-driver, was admitted to the institute at an inclusive charge of £2 2s. a week. Later on it was found that he was suffering from typhoid of a severe character, and the above claim was for brandy, eggs, and laundress. The judgment was hard upon the institute, as £2 2s. a week could not possibly cover the cost of nursing such a case. Nevertheless, it is only fair that a properly kept account of extras should be given to those who have to pay the bill, and we trust the institute will lay the lesson to heart.

### FAIR TO THE PROBATIONERS.

A point of the utmost importance to all young women entering the poor-law service is significantly marked in the case of the six probationers appointed to the Munster Workhouse Hospital. They are engaged for a period of three years at 3s. a week, and possibly hope to receive at the end of that time the position of a superintendent nurse. They labour under a delusion if this be so, for unless there be a resident medical officer and the workhouse hospital is a recognised training school they have no status, and cannot aspire to any higher office under the Poor Law than that of assistant nurse. We have several times called the attention of our readers to this fact, and trust that those who contemplate entering this branch of the nursing profession will be careful to ascertain if the institution at which they propose training is recognised by the Local Government Board for this purpose.

### SHORT ITEMS.

The Mother Superior of the Convent of Mary Immaculate, at Key West, has placed the convent and two school buildings at the disposal of the United States Government, and has offered the services of the nuns as nurses, which offer will be accepted in case of need.—The ball recently held in the Town Hall at Hanley, in aid of the Nursing Society, realised £34.—Southampton is losing one of its most popular Jubilee nurses; Miss Ellis is leaving to take up work in a private home in London.—The Burslem Nursing Association has lost its president and secretary by the death of Dr. J. Alcock. Dr. Alcock was for many years hon. surgeon of the North Staffordshire Infirmary and of the Haywood Hospital.—At the annual meeting of the Beith Nursing Association it was agreed to provide a bicycle for Nurse Macintosh.—The Royal Derby and Derbyshire Nursing and Sanitary Association possesses a staff of nurses numbering 65. Of these eight are district nurses and 12 probationers. There is a balance to the good on the year's accounts of £427.—A jumble sale was held on the 17th inst. in aid of the Bournemouth Jubilee Nursing Home.—The Brechin branch (N.B.) of the Victoria Jubilee Nursing Association is losing the services of Nurse Lyon, who for the last five and a half years has won golden opinions from all who knew her. She has resigned for private reasons, and is going to Edinburgh.—The Diamond Jubilee Committee of Bude has decided to divide £5 18s. 9d., the balance from a public tea, and £2 6s. 6d. from the Memorial Fund, amongst the Stratton Cottage Hospital, the Sick Nurses' Fund, and the Seamen's Shelter.—Nurse Cain died at Bombay from peritonitis on April 7th.—The sum of £912 has been handed over to the representatives of the Stockport Sick Nursing Institution as the proportion due from the Diamond Jubilee Celebration Fund.—The Prince and Princess of Wales will open the science laboratories at the London School of Medicine for Women on July 11th.

### Lecture to Nurses.

### ON PREPARATION FOR AND AFTER TREATMENT OF CASES OF ABDOMINAL SECTION.

By E. Stanmore Bishop, F.R.C S.Eng., Surgeon to the Ancoats Hospital, Manchester.

If ever to gain success it is necessary that surgeon and nurse shall thoroughly understand each other and work shoulder to shoulder, it is in cases of abdominal surgery, in which that cavity has to be opened, and if I have had any success at all in such surgery it has been because I have always had good, faithful, and trustworthy nurses to assist in and complete the work. My business to-night is to try and point out to you all, and especially to the later additions to our ranks, the principles upon which we have to work, and more particularly the details of such work with which you as nurses have to concern yourselves.

Now, the first principle, and perhaps the one which includes most of the rest is cleanliness. I mean surgical cleanliness, not the ordinary acceptation of the term, and I mean cleanliness, not merely of yourselves, but of your patients and of everything connected with them.

What is surgical cleanliness? It is freedom from micro-organisms. In the old days—I am speaking of thirty to forty years ago—surgeons operated as a rule in the dirtiest room in the hospital. I am old enough to remember when a surgeon carefully left his overcoat and coat in an ante-room, turned up his cuffs for fear of messing them, walked into the theatre up to a grimy wardrobe which was in one corner, and took out an old coat or dressing-gown which he had used for the same purpose for many years, splashed all over with blood, pus—and other things—and when all his spotless linen was well covered up in this, took his place at the table (an old wooden one) which matched his coat in the variety and gruesome character of its stains, prepared for any operation he might have to do. When all was over he washed his hands—it would have been an insult to have asked him to do so before—hung up his old coat with its fresh addition of blood on its accustomed hook, and rearrayed himself in clean coat and overcoat so that he might look smart and well-groomed outside. In those days the three necessaries for a surgeon were quickness, coolness, and accuracy, and his nurses, as was natural, matched him. They were quick, cool, and accurate, but no one ever thought of asking if they were clean. It would have been considered an insult. Because they were both clean—in the ordinary sense. They washed their faces and their hands at least twice daily; they very possibly bathed every day, or, at all events, as often as was in their opinion necessary. The surgeon always washed his hands carefully *after* his "dirty" work was done. The nurses had pretty clean gowns and natty wristbands, which have not gone out yet, and which I hope will not go out, for they are pleasant to see at the right time—but that is not in the operating theatre and during or before an operation. And their patients died of hospital gangrene, erisipelas, pyæmia, and septicæmia, or were interesting studies as to the various kinds of pus which it is possible to grow in a human body. There was laudable pus, ichorous pus, offensive pus, blue pus, yellow pus, grumous pus, and any other kind of pus you might have a scientific fancy for—and unfortunately there is still.

And this had been going on for centuries. Nobody thought anything of it, or that anything else was possible. Such a thing as primary union of a wound was known, but was looked upon as a curiosity, and gravely distrusted. The skin was believed to have an unlucky tendency to heal first, and to keep "secretions" pent up. These secretions were blood, lymph, serum, all ready and anxious to decompose, and by the products of their decomposition to inflame the wound and to necessitate its reopening, so that they might be "let out" like unruly schoolboys. Once they were let out it was necessary to plug the wound so that they might not again accumulate. Unfortunately, that is still sometimes necessary. Nobody knew why they decomposed. It was the "cussedness" of things, or, if anyone chose to go deeper, it was because, being out of the area of motion in vessels and capillaries, lymph and blood became dead foreign bodies, and therefore they decomposed. Of course, the "therefore" simply covered ignorance as to the true "why." But Pasteur and Lister, and others following their lines, by their researches cleared up this vital question. They showed by experiments, which I have only time to outline to you, that the changes produced were due to the presence and growth of external organisms, and that if it was possible to shut these out, or kill them when in, such changes did not occur. The outcome of these experiments was: (1) Animal fluids and tissues taken directly from the body by absolutely clean instruments, and preserved in absolutely clean vessels, might be kept for an indefinite time without undergoing any such changes as those of decomposition or fermentation. (2) The same fluids and tissues, if exposed to the air, or especially to the contact of dust or dirt, though kept in precisely the same way, promptly decomposed. (3) Portions of fluids and tissues introduced into the bodies of other animals, if this were done with perfect precautions against the inclusion of dust, dirt, or the admission of air, were absorbed quietly, and no inflammation was produced. (4) Portions of fluids and tissues, which had been exposed to air and had been contaminated in any way by dust and dirt, if introduced into other animals always produced inflammation, usually abscesses, sometimes death, death due to poisoning. (5) If a fluid which had been so exposed was filtered through cotton wool, it lost a great deal of its poisonous quality; this also occurred if it was mixed with certain drugs, notably carbolic acid. (6) In an infected fluid under the microscope with high magnifying lenses, small moving bodies were seen. In some cases these were mainly found in the scum floating upon the surface, in others in the sediment, which fell in time to the bottom of the test tube. (7) If some of these bodies were placed in a perfectly sterile fluid containing animal matter, they grew and multiplied enormously. (8) That some of these bodies, distinguishable by the size, shape, and in various other ways, if injected into living animals, always produced certain definite conditions, such as local abscess at the point of injection, whilst others, of a different size, form, &c., always produced other effects, such as death by general poisoning without abscesses, or before abscesses had time to form. Of course, at first, there was considerable confusion, as it was not well understood how these bodies were to be separated, and cases of "mixed infection" were the most frequent. But gradually this became more and more possible, and as it did so it became more and more evident that these bacilli, micrococci, microbes, whatever you like to call them, were not merely accidental accompaniments of those conditions, but were the veritable causes, so that now we can distinguish between a bacillus which will infallibly produce diphtheria, one which with equal certainty will produce cholera, one which is the certain cause of anthrax, a disease characterised by large carbuncular abscesses, and so on.

### CHANGE OF ADDRESS.

The address of the Camberwell District Nursing Association will in future be Knatchbull Road, Camberwell, S.E., and not Burton Road, Brixton, as heretofore.

### Hobbies and Accomplishments.

THE private nurse is by no means altogether equipped for her work when she obtains the certificate of her training school. There are many other accomplishments beyond those of her profession which will make her a success and vastly increase the happiness of her life in private work, for the social qualities of the private nurse are an important factor. Just as a guest in a country house is the more valued because she is the happy possessor of one or two hobbies and accomplishments which serve to amuse and interest her hosts and their friends on long dull wintry days, so too will that nurse be prized who can bring some resources of entertainment to the rescue of patients during a long convalescence. In lodgings, in hotels, at seaside or health resort, it is a great boon to the invalid if "nurse is so clever at knowing just how to pass an hour or two pleasantly."

The scope of nursing accomplishments is boundless. One very successful nurse in private practice is an adept at poker-work, and she literally charms away the convalescence of her patients by converting them into poker-work pupils. Young and old, masculine and feminine alike, her patients enter with zest into the decoration of blotting books and wooden bellows by means of the poker. She purposely uses only such simple designs as learners can easily acquire, and her patient-pupils declare their convalescent ordeal vanishes so pleasantly and quickly, their only regret is that they cannot pass through it all over again.

One young boy patient whom she nursed through the weary stages of a protracted case of phthisis became under her guidance a most accomplished poker-worker; and his friends speak with the utmost gratitude of the nurse who afforded him so delightful an occupation during these many months of illness. His specimens of work are highly prized by his family in memento of him and of his very happy last days—days which would probably have proved long and dreary only for his kind teacher.

Another nurse, mindful of long, dull days when weather conditions prevent the respite of even a few minutes in the open air, has gathered together a marvellous collection of the "penny wonders" of the London streets. The mechanism of these and their puzzle properties afford days of interested entertainment to her patients. Indeed, one noted statesman when she was nursing offered her a large sum of money for her interesting collection. But she was not to be tempted. She kept them in the interests of a posterity of convalescents.

Another simple method of whiling away the time and helping a patient to forget a continuous pain—such as that of facial neuralgia, &c.—lies in the game of patience. This proves fascinating to all classes of sick people, and some nurses are perfect walking encyclopædias of the art of "patience" playing. Many persons who consider cards as a more or less sinful temptation to the flesh feel no hesitation in playing the game of "patience"—a game which grows in attraction the oftener it is played. Indeed, many people to whom card-playing is almost abhorrent engage without hesitation or qualm of conscience in Japanese or Chinese games of the most gambling nature when these are supplied by nurse or friends. The fact that these are Eastern games robs them of their traditional wickedness! The art of wood and fret carving is a more ambitious form of accomplishment, but several nurses who have attended technical classes in these subjects—and such classes are very general throughout the country—have acquired at very trifling cost a hobby delightful and useful to themselves, and proving a great boon to the sick people under their care. Many patients requiring the services of a trained nurse for many months are very pleased to acquire at their nurse's hands the simple forms of fretwork carving. It is a hobby offering a wide and varied field of interest.

It is hardly necessary to mention that simple new kinds of fancy work, such as macramé, and netting, will form a bond of sympathy between nurse and patient. "Crazy patchwork," ugly as this may be, has pleasantly tided over many a long convalescence. One private nurse who collects autographs, and another whose hobby lies in postage stamps, find these afford interest to patients—and they often come across collectors of both among their patients—this circumstance placing them at once *en rapport*. Such nurses frequently help their patients to rearrange various albums, and so pass away in congenial occupation otherwise dull hours.

Several private nurses who have a taste for collecting and mounting flower specimens find this interest affords much pleasure to their patients in cases when convalescence takes them to a new part of the country where interesting botanical specimens are to be met with.

A knowledge of books, so as to be able to select the literature most suited to the taste and temperament of one's patients, as well as a talent for reading aloud will prove valuable to every private nurse. This article only sketches out a few of the talents which may help to bring happiness to patients and success to private nurses. The subject is a large one, and the hobbies and accomplishments suggested therein admit of wide and varied scope according to the taste and talents of the individual nurse. It would be interesting if nurses would bear testimony as to the particular hobbies which they have found most pleasing and acceptable to their patients.

### Nursing in Workhouses.

MISS GIBSON, Matron of the Birmingham Infirmary, read a paper on "Nursing in Workhouses," before the West Midland Poor-Law Conference, held recently at Malvern. In it she remarked that she feared the mere scientific side of the work was proving most attractive, and that nurses bestowed most attention on surgical and acute cases to the disadvantage of the aged infirm. She had studied the difficulty of obtaining nurses, especially in the smaller unions, both from the guardians and nurses' standpoints, and she did not see any prospect of their decreasing. The last order of the Local Government Board had not improved the situation, as the great majority of smaller unions had not a resident medical officer, and were not, therefore, recognised as training schools. In her opinion, the order might be amended. For instance, if infirmaries containing over 150 beds, having a trained superintendent nurse, and a visiting medical officer, gave three years' training and held examinations, the intention of the Local Government Board would be adequately fulfilled. She thought also that the absence of professional status, the low salaries and discomforts of surroundings in many workhouse infirmaries tended to increase the aversion that so many nurses evinced for poor-law work. The guardians ought also to uphold their nurses' authority. It was an abnormal position for a nurse to be under the control of an untrained matron. The question of class should not be brought into the matter at all. A matron might hold the better position, but as she was not a nurse, she ought not to control nursing. Miss Gibson threw out the following suggestion as to a way to meet the difficulty. Let there be a Workhouse Council, with its headquarters in London, and let each union infirmary undertake to train nurses for those next below it in importance. The lady guardians might select the candidates, and the board pay for their training in the selected infirmary, and require in return a period of five years' service. In the discussion that followed, Miss Gibson said that, in her opinion, eight patients were as many as one nurse could take charge of.

### Nursing in Paris Hospitals.

### B.—TRAINING LAY NURSES.

### III.—Practical Instruction.

As yet the weakest point in the training of the lay nurses is the practical training. The theoretical training is, of course, not so essential in ordinary duties, concerning, as it does, matters for which the medical and surgical staff are mainly responsible. The practical training is, on the contrary, an absolute necessity for the nurse from the day she enters upon her office.

With the probationers in each hospital such practical training as they receive is given by the assistants. Of course, these are not usually very highly qualified instructors, and the new schools are intended to offer something better. The schools, however, only affect a small proportion of the nurses, being confined to such part of the nurses in only four of the hospitals who follow the complete courses. It has been felt, however, to be so important to have a wider network of practical instruction, above all things, that the four schools have been supplemented by courses of practical instruction in four other hospitals.

The four hospitals thus favoured are the Cochin, the Necker, the Tenon, and La Charité. At the original institution of these supplementary schools in 1892 the Beaujon Hospital was selected instead of the Tenon Hospital. The idea did not seem, however, to flourish in this fashionable "west-end" institution, and the great modern hospital in the far east was substituted. Perhaps the fact that the director of Tenon, M. Amaury, is a very active up-to-date reformer, and is well acquainted with English and other foreign establishments, has something to do with the substitution. These supplementary courses have been carried on for some half a-dozen years past, but are somewhat irregularly attended. The report of the school year ending in July last gave the attendance at the Cochin Hospital as very creditable, being 12 day and 26 night female nurses, and 19 males, or 54 in all; and even this figure would have been larger if the Cochin hospital did not contain so many certificated nurses. On the other hand the attendances at the other three courses was absurdly small. At the Necker the lessons began in November with 22 women and 9 men, which fell in July to 13 women and only 3 men. This number is all the more inadequate when we call to mind that the staff of the great adjoining Enfants Malades furnished a share of the contingent. Moreover, for the course at Necker the instruction was given by a matron, and for the women by an assistant matron, and a male superintendent nurse gave a course in pharmacy, whereas at Cochin the course was begun by a single assistant matron, who was replaced in December by an assistant. At the great establishment on the summit of Menelmontant the course was more of a fizzle still, for although 25 of the Tenon nurses inscribed their names, only 8 really attended the lectures given by a certificated matron. Lastly, at La Charité, the course given by an assistant matron was followed by 12 male and 15 female nurses.

The most important feature in the system of practical instruction is that of rotation of service, the *roulement* as it is termed, for gaining experience in various sorts of cases in different hospitals. This was instituted to a limited extent in 1890 by M. Peyron, but there continues to be a demand for a further extension of the plan by the promoters of the schools. It is now practically confined to the nurses who have already obtained the certificates, but it is closed for those who are aspirants for the honour. Most of the transfers are made in the summer during the vacations, when the regular doctors and surgeons take their holidays, for the medical staff are great opponents to the frequent changes in the nursing staff.

The regular course of practical instruction in the schools consists of from twelve to twenty lessons given by a certificated matron, or assistant matron, in four sections—medicine, surgery, cupping, and baths (including vapour and shower baths). Among the special points emphasised in the nurses' manual of practical courses in medicine and surgery are "the care of cleanliness to be given to the sick, the steps to be taken in case of death, the recognition of the principal pharmaceutical substances, the handling of surgical instruments and appliances, the making of bandages, the taking of the temperature of the sick, the preparation of the beds, the keeping of the books, such as those of the linen, movements of patients, visits, &c." Complaint is made, especially in the case of male nurses, of backwardness in learning the all-important work of handling bandages. There has lately been instituted supplementary instruction in baby swaddling by certificated matrons from the *clinique d'accouchement*. The provisions for instruction in midwifery I will refer to in my special article on that subject. Also, Dr. Bourneville is never ceasing to agitate for instruction in asylum work and care of the insane, as he considers that in all great hospitals similar phenomena to regular lunacy have to be dealt with in the course of their duties by all nurses, and that all nurses should be instructed in at least the elementary knowledge of treatment of the insane.

Of course, the most important feature in the course of practical instruction is the annual examination to which the aspirants for diplomas are subject. This examination has shown a tendency to decrease in severity year by year. Whereas in 1891, at La Salpêtrière, it lasted for three days, last year and the year before the examination was generally restricted to one or at most two sittings in the four schools.

Encouragement has been given to all sorts of people connected with hospital administration—the butcher, the baker, and the candlestick maker—to attend both courses and examinations, and to strive to get diplomas, the idea being that anyone may be called upon for assistance in emergencies, and also that chances of promotion are thus opened to such qualified applicants.

Edmund R. Spearman.

### Presentations.

An interesting ceremony took place in the large dining hall of the Royal National Hospital, Ventnor, on Monday afternoon last, when Dr. Lewis was presented with a handsome travelling clock and illuminated address, bearing the names of 183 subscribers, being nearly the whole of the staff and patients at present at the hospital.

### The Epileptic Colony, Chalfont St. Peter.

On Thursday, May 12th, the Duchess of Marlborough opened the "Victoria House," the new home for men at the Colony, Chalfont St. Peter, Buckingham. On the same day the Duke of Marlborough laid the memorial stone of a home for boys, to be known as the "Milton" Home, and Mrs. Passmore Edwards the foundation stone of a home for girls. A large number of people assembled to witness the ceremonies, many being invited guests, for whose convenience a special train was run from Baker Street and carriages provided for the drive from Chorley Wood to the Colony, and on the return journey from the Colony to Rickmansworth.

After a short religious service, Mr. E. Montefiore Micholls, chairman of the Executive, gave an address of welcome in the temporary marquee erected near the Victoria House. He said that the Duke of York, who on a former occasion had become patron, had now promised to be president. This set a seal upon the value of their work, for, generously as the members of the Royal Family lent themselves to the encouragement of philanthropic enterprise, they did not identify themselves with an institution unless the objects were good and the work well carried out. It was five years since the Colony was founded through the generosity of Mr. Passmore Edwards, who bought the farm and gave it to them, and nearly every house upon it had been his gift. A promise of £1,000 to build a small hospital or sanatorium had been received from Mrs. Dearmer, and Mr. and Mrs. Frederick Green had given £2,000 to erect a house for men who can pay. This will enable the Colony to retain the services of a resident medical officer. The Colony has grown so rapidly that capital expenditure of large amounts have been necessary. The present drainage and water supply are both inadequate. The whole of the farm has been taken, and this has necessitated buying extra stock. The workshops needed enlarging, and there were other items. All these things trenched largely on the funds usually spent on general purposes. The matter, however, that caused them considerable anxiety at the present time was the furnishing of the new Victoria House. It would have caused a vast gap in their resources had they bought new furniture for it, consequently they had transferred the colonists, their goods and chattels from the temporary iron home into the permanent one. For heavy items of capital expenditure such as these, the committee looked to the generous donations of the wealthy. They wanted badly a central administration block, containing kitchen, &c., an engine for pumping water for cooking purposes, and to supply motive power for the electric light, for as yet the home was lighted by lamps, and although there had fortunately been no accidents, oil lamps were more or less dangerous, especially with the class of sufferers with whom they had to deal. The backbone, however, of charitable enterprise was always the annual subscriber to the maintenance fund, and the advent of such would greatly rejoice the heart of the committee. Mr. Micholls went on to say that England was behind the Continent and the United States in her provision for epileptics. In America a whole village had been requisitioned for their accommodation. But they were advancing with rapid strides, and were resolved to catch up those who were in front. He concluded with an apology for the untidiness caused by the building operations.

At this juncture the Duchess of Marlborough was presented with a bouquet of orchids, and Mrs. Passmore Edwards with one of pink roses and geraniums.

Sir William Broadbent then proposed a vote of thanks to the Duke and Duchess, and to Mr. and Mrs. Passmore Edwards. He also read a letter received that day from Sir Francis de Winton as follows: "The Duke of York wishes me to say how glad he is to hear of the progress of the Colony, and that he wishes it all success."

Dr. Dickinson, in seconding the vote of thanks, gave a short history of the treatment of the disease from the days when those afflicted with it were smothered. He discussed the three ways of dealing with epileptics—in prisons, in asylums, or in their own homes. There were, he said, two points of view—the domestic and the criminal. If uncontrolled, epileptics would frequently in their contrary moods be guilty of criminal acts for which they were not morally guilty, but of which the consequences were penal. They could not be treated satisfactorily in prisons where they were deprived of the kindness that ameliorated their sad lot, nor yet in their own homes. Affectionate relatives, by reason of their sympathy, were not able to exercise the discipline which was so essential. The only humane method of caring for these afflicted persons was to gather them into homes such as were provided by this colony. Dr. Dickinson said that these points ought to be made public, as by cutting off at the source a large number of persons who must, if neglected, eventually reach the prisons, they were rendering a national service, and were on this ground entitled to Government support.

The Duke of Marlborough returned thanks for the Duchess and himself, and said that politics were becoming more and more bound up with questions that affect the social amelioration of the people. As this became more widely recognised more credit would redound to those societies who, in improving the daily lives of the people in a way impossible to the cumbersome machinery of Government, rendered enormous service of inestimable value to our country. It gave him, he said, great pleasure to be allowed to take part in the day's ceremonies.

Mr. Passmore Edwards, after a few remarks, said that Milton had lived close to the colony, and that, if the committee would permit, he should wish the new boys' home to be named the "Milton" Home, and that one ward in the sanatorium to be built should be dedicated to the memory of Miss Frances Willard.

The Duchess of Marlborough was then conducted to the Victoria Home, where she was presented with a key. After unlocking the door, she pronounced the house open. The guests then proceeded to the boys'—the Milton—home. The Duke of Marlborough performed the ceremony of laying the memorial stone, and Mrs. Passmore Edwards afterwards laid the foundation stone of the girls' home. A silver trowel, suitably inscribed, was presented to each.

### Everybody's Opinion.

A CORRECTION.

The "Mother Superior," St. Peter's Home, Kilburn, N.W., writes: Having seen an announcement in your paper that we receive "incurable male patients" in this Home, I should be much obliged if you would insert a notice to the effect that we receive no male patients here at all. There is a ward at St. Michael's Home, Axbridge (of which we have the charge), for male consumptive patients, but otherwise we do not undertake the care of men in any of our nursing homes.

### Guy's Hospital Nurses' and Students' Concert.

The Guy's Hospital Nurses' Choral Society and the Students' Glee Club combined gave their second annual concert to quite a large audience in the Governor's Court Room on Tuesday night. The first part consisted of a cantata by J. B. Van Bree, "St. Cecilia's Day," in which the solo parts were taken by Miss Gomer Williams, and the choruses were excellently rendered by nurses and students. Part-songs and glees, songs by Miss Gomer Williams, Mr. J. McD. Parrott, and Mr. H. T. Hicks, which met with enthusiastic encores, a violin solo, and a musical sketch by Mr. Sydney Spalding, completed the programme, which was very much enjoyed by all who were present.

### A Book and its Story.

### A KING'S DIARY.

"A KING'S DIARY," * in Cassell's handy little series, the Pocket Library, is delightfully written, and is remarkable for the skilful manner in which its author unfolds his plot, and for the artistic restraint noticeable throughout its interesting pages. The story, which is autobiographical, opens with a description of a young man soliloquising on an all-engrossing study of himself—a young man, aged twenty-nine, who has fallen in love for the first time. "Come," he says, "let me look at my life. I am outwardly the same man I was three months ago, although inwardly all the furniture of my mind has changed. . . . In these chambers, near these dusty books, with the view across the river, and snorting of steam tugs, the whistle of locomotives always in my ears, for six years I have sat idly, alas! wondering what fate would bring me. Now it has brought me this! My Diary tells me I have met her twenty-five times. . . . I have not written her name yet—Christabel Fairfax." And further on he continues, in a more matter-of-fact tone: "Can I ask a beautiful girl to share my £300 a year, and my literary prospects? Literary prospects, forsooth! That little book of poems, 'Imitations of Catullus,' published at my own expense, and of which one hundred and fifty copies were sold, although the *Spectator* did say it was 'the work of a man of culture.' Now, if I could sing like a Shelley or a nightingale, I could not empty my full heart."

The young man's meditations terminate in a proposal of marriage to the object of his affections, the simple and lovely Christabel, whose father, Fairfax and Co., was a millionaire. Christabel's answer is in the affirmative. Archibald Seaton is invited to the family home in the country to receive the Fairfax reply. The approach to the house—a stately Queen Anne mansion—is through a curving drive in an ancient avenue of elms and beeches, "and up this avenue walked a briefless barrister to tell the owner he proposed to marry his daughter."

At the termination of the interview, Mr. Fairfax gives his consent to the engagement, on what grounds, besides the claims of the affection the young couple bore each other, it is hard to say. However, luck befell them, and Christabel's father arranges to allow her £800 a year, and Lord Linton, young Seaton's uncle, appointed him secretary to "The Depressed Landlords' Association," at which post the duties were light and the income some £200 a year. Archibald Seaton describes his wedding "that May morning," as he put it, in rapturous terms. "If the white and red hawthorn had taken human shape, and glided along the aisle of that country church, I should only have seen Christabel—a billowy white shape, with faint pink spots on her cheeks.

Eighteen months later Archibald's diary has the following entry: "My old bachelor life seems far back in the past. Christabel and I and our little boy five months' old are busy solving the problem of living together here in this pleasant house in the long street in Kensington." And he goes on to wonder if he is as absurdly sentimental as his wife infers he is; if he is impracticable, as his father-in-law considers he is; if he is "wanting in push," as his mother-in-law thinks he is. The entries are those of a man who is self-engrossed beyond the ordinary, whose analyses are morbid and unhealthy. Archibald Seaton has not the element of success; he loses his secretarial post, regarding which he admitted in his diary that his uncle declared "if he had played his cards properly the subscriptions would not have fallen off as they had done since his appointment." "For a man who cannot afford it, you are confoundedly independent," his uncle had commented on his nephew's indifference at the loss of the post.

*"A King's Diary." The Pocket Library. Percy White. (Cassell and Co., London.)

Meanwhile young Seaton sought for fresh employment through the medium of daily paper advertisements, which work he found on the staff of a new illustrated weekly journal, by name *The Demon.* This great enterprise, even in its embryo state, threatened to cut out all its contemporaries. Seaton is elected editor at a regular salary of £400 a year. Lady Finch, the most beautiful and fashionable woman in England—*vide* halfpenny evening papers—edits the Society Notes. *The Demon* is launched as a monster success, the question of finance alone standing in its way. It is arranged that the staff take shares. Seaton is led to believe that his appointment will be withdrawn unless he invests £2,000 in the promising venture. Regarding this, husband and wife have a consultation—Christabel, whose characteristic is a strong common-sense, demurs at the proposal—£2,000 is a large sum to advance. Seaton outweighs her arguments, and *The Demon* is enriched by the ill-spared £2,000. Husband and wife continue to feel an unexpressed resentment against each other—the subtle, indefinable misunderstanding which commenced at an earlier date in their married lives. Perhaps this is the most artistic point throughout this clever little story of Mr. White's—the analysis of the estrangement, wherein pathos and comedy play an equal part. Archibald felt his career was heroic and grand; Christabel was incapable of appreciating, and the gulf widened. Finally the two part company.

The reader's sympathies are with Archibald Seaton's misfortunes. He had missed success in life. His wife had ceased to love him. His financial position was hopeless—"The Demon" had failed to fulfil the great expectations raised for it. And at the crisis of his disasters, Christabel comes back to him, knowing all, and with a happy, radiant face, to stand by her husband in his evil hour.

Archibald's diary becomes bright once more, after Christabel's presence had lifted him out of the depths of despair. In the new heaven in which they find themselves, Christabel is summoned one day to a bedside in a hospital, to find her husband suffering from concussion of the brain, complicated by fracture of the skull.

The last chapter in Archibald Seaton's Diary, he writes: "I cannot remember when I was installed here in my palace, nor does it matter. I only know that they have made me King. . . . My rooms look over a wide garden . . . bound by high walls, that shut off my court from the world I rule beyond. I have abolished capitation, reconstructed society, and brought back the golden age to all save myself and—Christabel. Alas! for me there is no Queen. The Prince, my son, a golden-haired darling of five years, is the bond between us. Occasionally my Chamberlains bring them to see me. How often I cannot tell, for a great and lonely monarch marks not the flight of time. When the ceremonial is over my Royal content is banished for a while, and I am tempted to break my vows and return to the existence whence the loud voice of duty summoned me. Once I tried to abdicate, but my counsellors persuaded me to accept a kingly task."

In one of the visits of Christabel, she let fall a letter, which her husband read. Herein she describes in touching terms her husband's solitary confinement in the asylum. "His sole amusement now," the letter concludes, "is to write in his diary," and the last entry in the King's Diary, after he had read the above, runs: "Alas! poor Christabel, my Kingship is overthrowing your reason. But you could never rise to the height of self-abnegation required by the sharer of my throne. To you, 'the people' are but a name. To me—and unto the end I feel approaching—they must be the suffering multitude, whom I alone can save. . . . Their woes sap my life, and so I wither on this cold pinnacle of power—alone."

### Parents' National Educational Union.

I.

Some exceedingly interesting addresses have been given during the course of the annual Conference of the members of the Parents' National Educational Union, held in London during the past week. Amongst the speakers were: Miss Mason, the founder of the Union, Mademoiselle Duriaux, Miss McMillan, Mrs. Steinthal, Mrs. Spencer Curwen, Mrs. Franklin, the Hon. and Rev. Canon Lyttelton, Mr. H. B. Garrod, Mr. H. Geldart, Mr. T. G. Rooper, and Miss Blogg.

A paper read by Mrs. Steinthal, on "The Utilitarian Training of our Daughters," gave some practical suggestions on the encouragement of girls of all ages in habits of usefulness and philanthropy, and pleaded that girls should be trained to become useful heads of families, so that tales of domestic mistakes by young married women might no longer be heard Mrs. Steinthal told her audience of the "Order of Chivalry" started for children by Lord Winchelsea, each member of which is given a poor child as a "friend" to whom they write, and in whose concerns they are encouraged to interest themselves, saving their pocket money to give them little pleasures, and sending them flowers from the country. Then there was that difficult time in the lives of girls—the first two years after leaving school. In her own village Mrs. Steinthal had tried an experiment by starting a "Neighbours' Guild" amongst a number of young girls who undertook special sections, some doing needlework, others teaching embroidery to the members of a girls' club, or supplementing the work of the village nurse by visiting the sick, whilst there was an "entertainment section," the members arranging frequent concerts in the workhouses or convalescent homes around. Another guild, with similar objects, had been lately started in Sheffield. A feature was the "business drill," which the girls were now and again put through, when they learnt how to preside at meetings, and to put resolutions and amendments.

Mrs. Steinthal added that she did not forget that many girls left school thoroughly tired out, "brain-fagged," and these suggestions were not intended to burden them, but to help them to carry out Christian teaching, and to feel sympathy with their fellows, with the bad and vicious no less than with the good.

On Wednesday evening a conversazione was held at the Portman Rooms, at which the chief feature was an altogether excellent speech from the Hon. and Rev. Canon Lyttelton, the head master of Haileybury College, on "The Relation between Physical and Moral Training."

Replying to an allusion from the Chairman (Sir George Kekewich) to his fame in the cricketing field, Canon Lyttelton began by causing some amusement, while declaring his own love for the game of golf, in warning parents against it as a selfish game for the young, lacking in that spirit of co-operation which was essential if games were to do them good. Passing to the question of food, it was, he thought, a strange anomaly that food indulgence in old and young was looked at from such different points of view. Nearly all old people over-eat themselves, but when it was suggested that boys might do the same everybody shook their heads. Doctors agreed that boys wanted more food than men did, but it was surely possible to go too far in the crusade against what was called in the columns of the public newspapers "the gross insufficiency of food at all schools." Granted that boys wanted a great deal of food while they were growing, yet it was possible for them to be allowed to eat too much solid food. Twenty-nine out of thirty were brought up to consider that their appetites were never to be denied unless their pocket money ran short. If up to the age of 20 or 21 a young fellow had never known what it was to say "No" to his appetite, when he stopped growing this habit of indulgence would not cease, and in his view this was to foster the animal passions, a most serious question for all to consider. If the effect on the morals was to allow difficulties to arise in the way of young men leading wholesome lives, the responsibility was a grave one. Turning to what he would call "hardening" for boys, Canon Lyttelton put forward his conviction that a child should be brought up to believe that difficulties existed to be overcome, and he quoted a remark of the present Archbishop of Canterbury, a man who had himself been brought up on these lines. "You tell me," said the Archbishop, "that the thing is impossible; I tell you to make it possible by doing it." How many children in the present day were in this frame of mind? In his experience six out of seven simply refused to face difficulties, having been always helped over them, whereas they ought to come to school feeling that the difficulties were there to be got over. Canon Lyttelton went on to speak of the broad principle of the relation between matter and spirit, a subject which had enchained the attention of thoughtful people for centuries, and in connection with which he specially mentioned a book by Mr. Illingworth, "The Divine Immanence." Matter was coming to be regarded as the expression of the spirit, as inseparable from it; but the dignity of matter had been a thing much overlooked, and great harm was done by the idea that matter was in itself evil. Children should be made to think of their bodies as having a dignity of their own, and to realise their marvellously close connection with their personalities. It should be enforced upon them that the misuse of the body meant the misuse of the mind and the stunting of the spirit. Here was a deep and valuable lesson to be learnt, not confined to the narrow thing we call morals, but affecting the whole view of the universe.

### Minor Appointments.

Poplar and Stepney Sick Asylum, Bromley, Middlesex.—Miss Florence Mary Scott Cavell, who has lately been engaged in private nursing, was appointed Sister at this asylum on May 10th. Miss Cavell was trained at the Metropolitan Hospital, Kingsland Road, and holds a three years' certificate from St. John's House, 8, Norfolk Street, W. She was for some time charge nurse at the Fountain Hospital, Tooting.

Wolverhampton Union Workhouse Infirmary.—Miss Annie Campbell, who has been sister of St. Saviour's Infirmary, Dulwich, S.E., was appointed on the 13th inst. Superintendent Nurse of the above infirmary. She was trained at the Royal Surrey County Hospital, Guildford.

The Union Workhouse, Sevenoaks.—Miss Ada Thirza Morrell, who has held appointments under the Guardians of Paddington, Lewisham, Richmond, and Windsor, was appointed Nurse at the above on May 12th. She was trained at Paddington Infirmary.

Newton Abbott Workhouse Infirmary.—Miss Rosa Fisher, who was trained and who has worked in the Lambeth Infirmary, Brook Street, S.E., for eight years, has been appointed Superintendent of the Newton Abbott Workhouse Infirmary. She holds the L.O.S. certificate.

The Infirmary, Vallance Road, Whitechapel.—Miss Emily Phillips was appointed Superintendent Night Nurse here on May 3rd. She was trained at Leicester Infirmary, and has been nurse at the Glasgow Maternity Hospital and nurse at the Hospital for Women, Soho Square.

Inverness District Asylum.—Miss Bella McKenzie, who for two years has had charge of the Female Hospital, Royal Lunatic Asylum, Dundee, has been appointed Charge Nurse to the Male Hospital, Inverness District Asylum.

Rochdale Infirmary, Lancashire.—Miss Annie Tanner was appointed Charge Nurse of the above infirmary on April 21st. She was trained at the Bradford Royal Infirmary, where she gained the gold medal for 1898.

Isle of Thanet Union Infirmary.—Miss Ada Boulding, who was trained at the Toxteth Infirmary, Liverpool, has just been appointed Nurse of the above infirmary.

### The Duel in Germany.

THE medical student of to-day, as seen by the hospital nurse, is a very different being from what he was in the days of Albert Smith. He is respectable—we had almost said he is smug—and, in the wards at any rate, he is curiously amenable to discipline. The romance of student life has gone out, and "walking the hospitals" is no longer regarded by proud aunts and admiring sisters as a career of wild adventure. In fact, the English student, if he wishes to make display at all, has, like other men, to show his prowess in the cricket or the football field. Happy then is the school which has a good recreation ground, for without such opportunity for manly sports the medical student of to-day tends, much to his detriment, to degenerate into a tame cat and a sipper of much tea. It is somewhat different in Germany, where the duel still remains an institution, and a man who wishes to maintain his position must be prepared to fight. In a recent number of the *Guy's Hospital Gazette* a very full account is given by an eye-witness of the way in which the duel is conducted in Germany, and from it we extract the following, which, we think, will be of interest, especially in view of the final paragraph concerning German womankind.

"The men being dressed, march in two little processions into the larger room. These consist of the principal, whose right arm is supported by a student, extended on a level with the shoulder to prevent fatigue from the weight of his padded sleeve. His second, guarded as to head, eyes, throat, and right arm, and armed with a blunt schlaeger. Two doctors, one of whom carries the sharp schlaeger, the end of which has been rendered aseptic, and the dresser; this last is like our ground man, and his only function is to remove or replace the principal's chair. These contests are wrapped in so much formality that an account of many would be uninteresting. I'll try and detail one, the most bloody and the longest that I witnessed.

"Six men from 'Saxonia,' the crack corps of Tübingen University, had come over to fight with 'Leonensia' of Heidelberg. The president of this latter corps is the reputed best fencer here, and a son of Dr. Czerny, the eminent surgeon. The presidents of each corps were to fight together, the others balloting for their opponents. The two men, much of a size as far as one could judge, take their places, pale, but with a fierce look about them which their goggles may have exaggerated. The umpire seats himself. The seconds uncover, and state in turn the name and standing of their principals. The umpire announces the duration of the duel (generally fifteen minutes, which can be prolonged to half an hour), and then orders the "Ehrengang," or salute. The principals step sharply forward to within a schlaeger's length of each other, standing with feet apart, the right a little in advance, the right arm fully extended above the head, inclined slightly to the left. One of the seconds gives forth a prolonged yell, which I discovered afterwards was "Auf Mensur, fertig, los" (To the fight, ready, go), and the schlaegers wheel round in salute to return "on guard." The umpire again calls "Silentium," and orders the first round. Principals fix themselves firmly in the sawdust, and the seconds bend back sideways (they are on the left of their men), the shout is repeated, and strokes are exchanged with great rapidity, "parry and riposte," with no interval. After eight or a dozen strokes are exchanged the seconds cry halt, and interpose their weapons. No wound has been received; this they announce to the umpire, who orders a renewal. Again the yell. This time the contestants seemed to have gauged each other's powers. There is a little pause before commencement. Suddenly, whiz, and a wisp of hair flies into the air from one man's head; then blows in rapid succession. A yell of "halt," and a little red line on the forehead seen and heard at the same time. This is examined by the doctor, who dabs it with a plug of wool dipped in hyd. perchlor., and steps back; it is announced to the umpire by the second, and is noted down—first blood falls to Heidelberg. Tübingen tightens his lips and looks like business, which he duly effects, for before the next cry of halt he manages to score a three-inch cut on his adversary's scalp. A longer halt this time, then at it again. Both men have now got their blood up and seem to put more muscle into their strokes. A red line appears on Heidelberg's nose, and Tübingen's forehead cut is continued at an angle down over the left eye. On the renewal the men present a ghastly sight; both are pale, the blood freely oozing from their wounds, running down over their faces and cuirasses, and dripping on to the sawdusted floor. The last round is announced. The men are about equally much cut about, so both are keen on getting in a long many-suture-requiring stroke. This, after a particularly savage exchange of blows, falls to Tübingen. Parrying very rapidly, he follows with a cut which gets in under Heidelberg's guard, and a long line appears on the latter's cheek; it widens quickly, shooting out little spurts of blood near the ear, then pours down over the face. The doctor steps up quickly, looks grave. Heidelberg gets rapidly paler, and finally sinks into his second's arms. Tübingen shakes him limply by the hand, and both men are assisted out. This man's temporal artery was severed, and Heidelberg's scalp wound went through scalp and periosteum down to bone. . . .

"The womankind here in Germany are, in a measure, responsible for the continuance of the system, as they dearly love to see a few scars on a man's face. It denotes the possession of a certain amount of pluck, and shows that the man is not only a bookworm. Here, for instance, and Heidelberg is very representative, there are 1,500 men, and of these only about 300 fight. For this reason, perhaps, men seek a scar or two. It is most unusual for a fighting man to get through his university life unscarred—Bismarck was one. An Englishman who was here a few years ago held the record number of duels, and left unscarred."

### Where to Go.

ROYAL BRITISH NURSES' ASSOCIATION.—At the rooms of the Royal Medical and Chirurgical Society, 20, Hanover Square, on Tuesday, May 24th, at five p.m., a special meeting of the Royal British Nurses' Association, to formally approve of the alterations suggested by the Lord of the Privy Council in the bye-laws passed by the general meeting on December 17th, 1897. H.R.H. Princess Christian, the president, has intimated her intention of being present. Copies of the bye-laws have been sent out with the May number of the *Nurses' Journal*.

THE DOWDESWELL GALLERIES, 160, NEW BOND STREET, W.—Nurses and others who have had their interest aroused in the East should not fail to visit these galleries, where Mr. Hampson Jones's charming water-color sketches of Ceylon and Burma are now on view. The collection is entitled "A Trip to the East." Munkacsy's great picture, "Ecce Homo," is still on view at the same galleries. Between 30,000 and 40,000 have visited it since January last.

A DAY OF REST AT HERTINGFORDBURY.—Metropolitan nurses are offered the opportunity of spending a quiet day in Hertford for the moderate and inclusive sum of 2s. 6d. Three short services, at each of which an address will be given by the Bishop of Thetford, will be held in the parish church. Those who wish to be present should communicate with the Rev. A. B. Locke, St. George's Hospital, S.W., or with the Rev. Canon Burnside, Hertingfordbury, Hertford.

AT the Hanover Gallery, 47, New Bond Street, M. Paul Boyer is showing a large selection of photographs, which have been taken under a new process of his invention. He claims that an instantaneous exposure with artificial light, occupying the one-hundredth part of a second, will produce directly photographs of any size, surpassing those which are taken in daylight.

### For Reading to the Sick.

ASCENSIONTIDE.

"I ASCEND unto My Father, and your Father; and to My God, and your God."—S. John xx. 17.

**Verses.**

There for Him high triumph waits;
Lift your heads, eternal gates;
He hath conquered death and sin;
Take the King of Glory in. Alleluia!
See! He lifts His hands above;
See, He shows the prints of love;
Hark! His gracious lips bestow
Blessings on His Church below. Alleluia!
—*Charles Wesley.*

Right gloriously He triumphs now,
Worthy to Whom should all things bow;
And joining earth and heaven again,
Links in one commonweal the twain.

And we, as those His deeds we sing,
His suppliant soldiers, pray our King,
That in His Palace, bright and vast,
We may keep watch and ward at last.
—*S. Fulbert of Chartres.*

See the Conqueror mounts in triumph,
See the King in Royal state
Riding on the clouds His chariot
To His heavenly palace gate.
Hark! the choir of angel voices
Joyful alleluias sing,
And the portals high are lifted
To receive their heavenly King.
—*Bishop Chr. Wordsworth.*

To whom the angels drawing nigh,
Why stand and gaze upon the sky?
This is the Saviour, thus they say
This is His noble triumph day.
Again shall ye behold Him, so
As ye to-day have seen him go,
In glorious pomp ascending high
Up to the portals of the sky. —*Latin Hymn.*

**Reading.**

Truly, if we could ever live in this day, all were joy. It is the crown of all joys, the joy of all creation, the wonder of the blessed angels, the union of all being, the finishing of the earthly course of the Son of God, His entrance into glory. He ascended not into the highest heavens only, but far above all heavens. There, where no creature is or can be; there encircled, embosomed, impenetrated with the Godhead, adored altogether with His Godhead by all creation, is the Body of Christ, our God, our King, our Head; Who calls us "His Body," "calleth us brethren."—*Dr. Pusey.*

It is the duty and privilege of all disciples of our glorified Saviour to be exalted and transfigured with Him; to live in heaven, in their thoughts, motives, aims, desires, likings, prayers, praises, intercessions, even while they are in the flesh; to look like other men, to be busy like other men, to be passed over in the crowd of men, or even to be scorned or oppressed, as other men may be, but the while to have a secret channel of communication with the Most High—a gift the world knows not of, to have their life "hid with Christ in God."—*Dr. Newman.*

### Wants and Workers.

NURSE ASHBEE, care of M. Hawthorn, Esq., Avondale, Wallington, Surrey, would be glad to buy a secondhand copy of Sparkes' "Artistic Anatomy," and of Furmany's "Elementary Physiology."

### Notes and Queries.

**The contents of the Editor's Letter-box have now reached such unwieldy proportions that it has become necessary to establish a hard and fast rule regarding Answers to Correspondents. In future, all questions requiring replies will continue to be answered in this column without any fee. If an answer is required by letter, a fee of half-a-crown must be enclosed with the note containing the enquiry. We are always pleased to help our numerous correspondents to the fullest extent, and we can trust them to sympathise in the overwhelming amount of writing which makes the new rules a necessity. Every communication must be accompanied by the writer's name and address, otherwise it will receive no attention.**

*Swedish Movements.*

(68) Kindly tell me the name and publishers of a book on Swedish movements, and also on the Nauheim treatment.—*Inquirer.*

The Scientific Press, London, can supply "Massage and the Original Swedish Movements," by K. W. Ostrom, for 3s. 6d. net, and "The Schott Methods of the Treatment of Chronic Diseases of the Heart," by Dr. Bezly Thorne; or "The Schott Treatment for Chronic Heart Diseases," by Dr. Richard Greene, price 6d.

*As Probationer.*

(69) I have asylum training and wish to become a probationer. Where can I obtain work and wages?—*C. P.*

See Honnor Morten's "How to Become a Nurse," 2s. 6d. (The Scientific Press, London); or see our advertisement columns.

*Stature.*

(70) Will my not being tall be any hindrance to my becoming a nurse? I am very strong and healthy.—*Constant Reader.*

Certainly not.

*The Nurses' Bookshelf.*

(71) I wish to become a nurse. Please tell me what kind of books to read whilst waiting to enter a hospital also the price and how to obtain them?—*M. C.*

"Nursing: Its Theory and Practice," by Lewis, 3s. 6d. net; "A Manual of Hygiene for Students and Nurses" by John Glaister, 3s. 6d. (The Scientific Press, London). Why not study cookery? It is a most important branch of the nurse's art.

*Theoretical Midwifery Training.*

(72) I have been superintendent nurse in workhouses, and have constantly attended midwifery cases. I now wish to take the certificate of the L.O.S. Must I begin my training all over again as a student?—*Anxious One.*

There is no necessity to do so. Get the medical officer of one of the workhouses to give you a written certificate that you have attended twenty-five cases, and then enter for the theoretical portion of the course. If you require coaching Miss Lamport, 52, St. John's Wood Road, N.W., teaches by correspondence, and at the Midwives Institute, 12, Buckingham Street, Strand, you may join classes for a course of lectures to prepare you for the examination.

*South Africa.*

(73) Is there a good opening for a private nurse in South Africa? Kindly give me the name of a hospital.—*Salopian.*

There is no greater demand for nurses there than elsewhere, and a private nurse should go out to definite work. South Africa is a large country. The New Somerset Hospital, Cape Town, and the Kimberley Hospital are among the most important of the hospitals.

*Probationers' Travelling Expenses.*

(74) Is it customary to pay probationers' travelling expenses? If I engaged a nurse-probationer in London, and she left at the end of the month, should I have to pay her fare both ways?—*Fever Matron.*

As it is not unusual for probationers on trial to deposit a sum with the matron to cover her expenses, which sum is returned when the agreement is ratified, you would be perfectly justified in making an arrangement that the probationer coming on trial should pay her own travelling expenses. But you must make it definitely in writing before engaging her.

*St. John the Divine.*

(75) I desire to become a trained nurse for district work. Kindly give me the address of the Nursing Sisters of St. John the Divine, and how I could enter it.—*R. T.*

Apply the Sister Superior, 19 and 21, Drayton Gardens, Kensington, S.W.

*Disinfecting after Measles.*

(76) In nursing children with measles, how shall I best keep in good condition the polished floors of the two rooms they occupy? At present I wipe them over daily with Sanitas in water. How shall I preserve the polish? When the children have recovered ought I to disinfect a baize door between the two rooms, and if so, how?—*Mother.*

Beeswax and turpentine well rubbed in not only keeps the floor bright, but prevents it absorbing infection. After the disease is over scrub everything, baize door included, with soap and water. The floor will soon polish again, and the baize take no harm if the water used be not too hot, and the soap lather well rinsed out.

*Maternity Work.*

(77) Can you tell me of an institution in London where they take trained nurses to learn maternity work free of charge?—*M. Ll. W.*

There is none that we know of.

**ANSWERS REQUESTED.**

*Home for Inebriates on the Continent.*

(78) Are there any homes for inebriates on the Continent? If so, where could I obtain particulars of same?—*M. W.*

